# Skull fracture and haemorrhage pattern among fatal and nonfatal head injury assault victims – a critical analysis

**DOI:** 10.5249/jivr.v2i2.46

**Published:** 2010-06

**Authors:** Saurabh Chattopadhyay, Chandrabhal Tripathi

**Affiliations:** ^*a*^Department of Forensic Medicine, Bankura Sammilani Medical College, Bankura, West Bengal, India.; ^*b*^Department of Forensic Medicine, Institute of Medical Sciences, BHU, Varanasi, India.

## Abstract

**Background:**

The global incidence of fatal head injuries as the result of assault is greater than the number of non-fatal cases. The important factors that determine the outcome in terms of survival of such head injury cases include the type of weapon used, type and site of skull fracture, intra cranial haemorrhage and the brain injury. The present study aims to highlight the role of skull fractures as an indirect indicator of force of impact and the intra cranial haemorrhage by a comparative study of assault victims with fatal and nonfatal head injuries.

**Methods:**

91 head injury cases resulting from assault were studied in the Department of Forensic Medicine, IMS, BHU Varanasi over a period of 2 years from which 18 patients survived and 73 cases had a lethal outcome. Details of the fatal cases were obtained from the police inquest and an autopsy while examination of the surviving patients was done after obtaining an informed consent. The data so obtained were analyzed and presented in the study.

**Results:**

Assault with firearms often led to fatality whereas with assault involving blunt weapons the survival rate was higher. Multiple cranial bones were involved in 69.3% cases while comminuted fracture of the skull was common among the fatal cases. Fracture of the base of the skull was noted only in the fatal cases and a combination of subdural and subarachnoid haemorrhage was found in the majority of the fatal cases.

**Conclusions:**

The present study shows skull fractures to be an important indicator of severity of trauma in attacks to the head. Multiple bone fracture, comminuted fracture and base fractures may be considered as high risk factors in attempted homicide cases.

## Introduction

Inflicting injury to the head is one of the most effective methods of homicide. The recent rise in the trend of murder cases involving head injury is a serious concern to society. Assailants usually select a part of the body where the maximum damage can be inflicted with minimum effort. The ultimate outcome of the attempt depends on a number of factors e.g. type of weapon used, target site on the body, number of blows etc. The presence or absence of a skull fracture, its type and site along with the type of intracranial haemorrhage has immense significance in the final outcome in cases of such head injuries. The thickness of the cranium is not uniform all throughout as there are thin plates of bones such as the frontal and temporal plates and the thickness is greater along the sutures.^1^ The greater the force of impact, the greater will be the damage and the more lethal the outcome. The present study aims to establish this fact by means of examining skull fractures which are an indirect indicator of severity of trauma. The type and the extent of the intra cranial haemorrhage also play a vital role in determining the ultimate result. Hence the chances of survival for a victim of these head injuries will be determined to a large extent, apart from other factors, on the intensity and effect of the injury on the skull and the brain itself.

## Methods

A prospective study of 91 cases of head injury resulting from assault was conducted at the Department of Forensic Medicine, Institute of Medical Sciences, Banaras Hindu University, Varanasi, India and S.S. Hospital Varanasi, India over a period of two years. Among these 73 cases proved to be fatal while 18 cases survived. These 18 patients can be considered to be victims of attempted homicide. All the cases suffered head injury due to an attack, with or without injuries to other parts of the body. The cause of death in the fatal cases was solely due to head injury and all cases where death occurred due to other injuries or other injuries contributed to the fatal outcome were excluded from the study. Nine cases died in hospital and the rest either died on the spot or on the way to the hospital. Information regarding the victims was collected from the inquest report, interviewing the family members and a proper and detailed autopsy examination in fatal cases and from the clinical examination and hospital records in nonfatal cases. Plain / non-contrast CT scan of the head was performed on all patients admitted to hospital to detect the intra cranial injuries. Statistical analysis was done using the chi square test and the p value was calculated.

## Results

**Fatal cases**

Among the fatal cases, firearms were the most common assault weapon and were used in 43.9% of cases followed by blunt instruments 34.3% (n=25). Sharp weapons were the least common weapon [Table 1].

**Table:** Table 1: **Type of weapon used**

Type of weapon	Fatal Cases(%)	Non Fatal Cases(%)	Total Cases(%)
Only blunt weapon	25(34.3)	13(72.2)	38(41.7)
Only sharp weapon	8(10.9)	-(-)	8(8.8)
Blunt and sharp weapons	8(10.9)	4(22.2)	12(13.2)
Firearms	32(43.9)	1(5.5)	42(36.3)
Total	73(100)	18(100)	91(100)

Comminuted fracture of the skull was observed in nearly half (49.3%) of the fatal cases[Table 2] and multiple cranial bone fracture was detected in over three quarters (76.7% n= 56) of the cases [Table 3]. Comparing the multiple and single cranial bone fractures among the fatal and nonfatal cases and applying the chi square test, multiple bone fracture was found to be highly significant for fatality p = 0.0005.

**Table:** Table 2: **Type of skull fracture**

Type of skull fracture	Fatal Cases(%)	Non Fatal Cases(%)	Total Cases(%)
Only fissure	10(13.6)	10(55.5)	20(21.9)
Comminuted	36(49.3)	3(16.6)	39(42.9)
Depressed comminuted	-(-)	2(11.2)	2(2.2)
Fissure + depressed comminuted	7(9.5)	2(11.2)	9(9.9)
Fissure + Sutural diastasis	3(4.2)	-(-)	3(3.3)
Firearm + Fissure	12(16.5)	-(-)	12(13.2)
No fracture	5(6.9)	1(5.5)	6(6.6)
Total	73(100)	18(100)	91(100)

**Table:** Table 3: **Site of skull fracture**

Site of skull fracture	Fatal		Non Fatal		total	
	Cases	%	Cases	%	Cases	%
Only frontal	4	5.4	3	16.7	7	7.7
Only parietal	3	4.2	5	27.7	8	8.8
Only temporal	2	2.8	2	11.1	4	4.3
Only occipital	3	4.2	-	-	3	3.4
Frontal + Parietal	6	8.2	3	16.7	9	9.8
Frontal + Temporal	11	15.1	1	5.6	12	13.2
Parietal + Temporal	10	13.6	3	16.7	13	14.3
Parietal + Occipital	2	2.8	-	-	2	2.1
Temporal + Occipital	7	9.5	-	-	7	7.7
Frontal + Parietal + Temporal	17	23.2	-	-	17	18.8
Parietal + Temporal + Occipital	3	4.2	-	-	3	3.4
No Fracture	5	6.8	1	5.5	6	6.5
Total	73	100	18	100	91	100

**Fig 1: Fracture base of skull:**
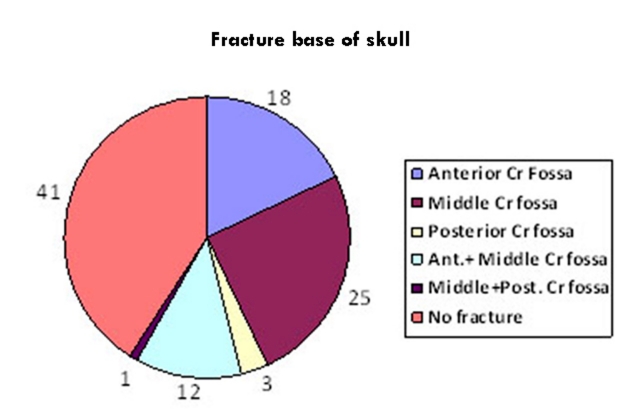
Fracture base of skull

In our study, fracture of the base of the skull was found only among the fatal cases (58.9% n=43). Middle cranial fossa was the most vulnerable to mechanical injury (38.35% n=28) while fracture of the posterior cranial fossa was the least common (4.1%) [Fig1].

Subdural and sub arachnoid haemorrhage together were found in 61.6% (n=45) cases [Table 4].

**Table:** Table 4: **Type of intracranial haemorrhage**

Type of intracranial haemorrhage	Fatal		Non Fatal		total	
	Cases	%	Cases	%	Cases	%
Only epidural	7	9.5	6	33.34	13	14.3
Only subdural	4	5.5	-	-	4	4.3
Only subarachnoid	5	6.8	1	5.55	6	6.5
Epidural + subdural	4	5.5	3	16.67	7	7.7
Epidural + subarachnoid	2	2.7	4	22.23	6	6.5
Epidural+ subdural + subarachnoid	10	13.7	1	5.55	11	12.3
Epidural + subdural + subarachnoid + intra cerebral	5	6.8	-	-	5	5.5
Subdural + subarachnoid	17	23.3	1	5.55	18	19.8
Subdural + subarachnoid + intra cerebral	13	17.8	1	5.55	14	15.4
Subarachnoid + intra cerebral	3	4.2	1	5.55	4	4.3
No haemorrhage	3	4.2	-	-	3	3.4
Total	73	100	18	100	91	100

**Nonfatal cases**

The most common assault weapons were blunt objects 72.2% [Table 1]  which resulted in fissure fracture as the commonest type of fracture 55.5% [Table 2]. Single cranial bone fracture was noted among the majority (55.5%) of the survivors [Table 3]. Epidural haemorrhage either alone or in combination with another type of haemorrhage was noted in 77.7% (n=14) cases [Table 4].

## Discussion

In the present study 80.2% fatality rate was noted among the assault victims. Increase in the use of firearms in cases of assault has been a recent trend. Previously blunt and sharp weapons were commonly used as reported by Mohanty et al,^2^ Mittal et al,^3^ Gupta et al,^4^ Aggarwal et al,^5^ and Dikshit et al,^6^ Pal et al,^7^ found the use of blunt weapons in 48.3% cases with the head and face as the primary targets. The present study clearly shows the preponderance of the use of firearms and their effectiveness as a murder weapon. Memchoubi PH et al,^8^ also reported firearms to be the most common murder weapon in their study. Such an increasing trend was already pointed out by Sagar et al,^9^ and Pollock et al,^10^ Fingerhut et al,^11^ also reported firearms to be the most common murder weapon in the United States. Being light, handy and easy to use, they are far more effective than blunt objects or sharp weapons. Chances of survival following blunt trauma to the head is far greater as indicated in our study. Sharp weapons are also not as effective as a means of inflicting head injuries compared to inflicting injuries to the chest or abdomen as it is difficult to penetrate the cranial bones.

Our study also showed that comminuted fracture of the skull was the commonest type of skull fracture among the fatal cases. In other words it can be said that the chances of fatality is greater with comminuted fracture than with fissure fracture. The damage to any tissue by mechanical force depends on the amount of energy absorbed by the tissues. Yavuz et al,^12^ in their study showed that the occurrence, degree of deformation and extent of fracture is related to the striking power, area of strike and physical properties of the skull at the point of impact. Presence of a comminuted or depressed comminuted fracture indicates the application of a great amount of force as compared to a fissure fracture. The greater the force used, the greater is the force transmitted to the underlying brain causing damage.

The chances of fatality further increase when firearms, as noted in our study, produce comminuted fractures. Thali et al,^13^ in their experimental study of gunshot wounds to a skull-brain model documented the dynamic creation of skull and brain wound morphology. In such cases, not only there is the effect of the forces being transmitted to the brain but also the penetrating bullet causes extensive damage to the brain along with the effect of cavitation. In depressed comminuted fractures produced by blunt or sharp weapons, fragments of bones impinge on the underlying brain and cause damage to the vital centres leading to death. Hence the type of weapon used has a significant role to play in the final outcome. Among the cases where firearms were used, 20 cases revealed comminuted fractures whereas when blunt weapons were used the force was much less resulting in a fissure fracture and ultimately the victims survived.

Our study clearly points out to the fact that the chances of fatality following head injury are greater when multiple cranial bones are involved p=0.0005. Overall in 69.3% cases multiple bone fracture was detected. On the other hand, 10 out of the 18 survivors (55.56%) had only single bone fracture which indicates that the force of impact was less. This also indicates the degree of force applied to the head. As the firearms were the commonest weapon used, the force on impact was maximum which was transmitted to the adjacent bones resulting in multiple bone fracture. The formation of radial fracture result in the release of circumferential hoop stresses induced by the bullet and concentric heaving fracture may result if additional stress release is required.^14,15^ Frontal, parietal and temporal bones were the site of fracture in most of the cases. Yavuz et al,^12^ also reported linear fractures to be most common in the frontal and temporal region. The occipital bone showed the least involvement in fracture, as it is the thickest among the cranial bones. Fracture of the occipital bone requires a great amount of force, hence when the occipital is fractured the magnitude of the force is considerable and sufficient to cause death in the ordinary course of nature.

Fracture of the base of the skull is associated with high fatality. As the vital centres are situated in the midbrain and brain stem, if the force of impact is transmitted to the base thus causing fracture, it is likely to cause damage to the vital centres. Moreover any haemorrhage in this area is likely to compress the vital centres. The anterior and middle cranial fossa was fractured in 54.8% (n=40) of the fatal cases. This can be explained from the fact that as most of the fractures in the vault were noted in the frontal and temporal bones hence the force was transmitted along these bones to fracture the anterior and middle cranial fossa. Moreover the anterior cranial fossa consists of the thin orbital plate and the cribriform plate, where chances of fracture are greater.

The occurrence of intracranial haemorrhage is very closely related to fracture of the skull. In our study only in 3 cases was there no intracranial haemorrhage. Epidural haemorrhage was more common among the non fatal cases. On the other hand subdural or subarachnoid haemorrhage either alone or in combination with other types of haemorrhage was more common among the fatal cases. Hence it can be inferred that the deeper the haemorrhage in the cranial cavity, the greater are the chances of fatality. This fact is further strengthened by the findings that intra cerebral haemorrhage was detected in 28.8% (n=21) of the fatal cases. On the other hand there was no significant difference in the mortality when considering whether the haemorrhage was located in multiple layers of meningeal space or in a single meningeal space p=0.16. Srinivasan^16^in his study reported CT scan to be one of the important predictors of head injury. Though in our study we relied on CT scan as there was no access to MRI, it should be noted that minimal shear bleedings may be better detected by MRI. Moreover in subacute and chronic stages MRI is superior to CT in detecting haemorrhages.^17^

The increased frequency of epidural haemorrhage in the nonfatal cases is due to the fact that in most of the cases there was blunt force impact over the parietal and temporal regions leading to fissure fracture of the bones, which ultimately lead to tearing of the blood vessels in the epidural space. On the other hand among the fatal cases there was multiple bone fracture. Hence the force of impact was more, and this was transmitted to the deeper tissues in the brain leading to subdural, subarachnoid and intra cerebral haemorrhage. Yavuz et al. [12] also reported in their study that epidural and subdural haemorrhages were more common with linear fractures whereas with depressed fractures laceration and contusion of the brain was more common. Gennarelli et al,^18^ and Lobato et al.^19^ have shown that the type of lesion is as important as the severity of injury when determining the outcome of head injury cases.

## Conclusion

Fatality among attack victims with firearm injuries to the head is very high. The type and site of skull fracture and the number of cranial bones involved is an indirect indicator of the severity of force of impact which leads to damage to the underlying brain and results in fatality. The location of meningeal haemorrhage whether in single or multiple layers has little influence on the outcome while haemorrhage in the deeper layers has higher fatality. Thus these may be considered as high risk factors in violent attacks to the head.
